# Magnetic resonance imaging-guided linear accelerator arterial spin labelling reveals dynamics of highly perfused non-enhancing glioblastoma during radiotherapy

**DOI:** 10.1016/j.phro.2025.100870

**Published:** 2025-11-21

**Authors:** Liam S.P. Lawrence, Brige P. Chugh, James Stewart, Mark Ruschin, Aimee Theriault, Jay Detsky, Pejman J. Maralani, Chia-Lin Tseng, Hany Soliman, Mary Jane Lim-Fat, Sunit Das, Arjun Sahgal, Angus Z. Lau

**Affiliations:** a Medical Biophysics, University of Toronto, Toronto, Ontario, Canada; b Department of Radiation Oncology, Sunnybrook Health Sciences Centre, University of Toronto, Toronto, Ontario, Canada; c Department of Physics, Toronto Metropolitan University, Toronto, Ontario, Canada; d Medical Imaging, University of Toronto, Sunnybrook Health Sciences Centre, Toronto, Ontario, Canada; e Division of Neurology, Department of Medicine, Sunnybrook Health Sciences Centre, University of Toronto, Toronto, Ontario, Canada; f Department of Surgery, St. Michael’s Hospital, Toronto, Ontario, Canada; g Physical Sciences Platform, Sunnybrook Research Institute, Toronto, Ontario, Canada

**Keywords:** Glioblastoma, Arterial spin labelling, MRI-linac, Perfusion, Repeatability, Tumour dynamics

## Abstract

**Background and Purpose:**

Targeting tumour vasculature during radiotherapy is a direction of current investigation for radiotherapy strategies in glioblastoma. Arterial spin labelling (ASL), a perfusion imaging technique that uses arterial water as an endogenous tracer, on magnetic resonance imaging (MRI)-guided linear accelerators (MRI-linacs) could guide adaptation to changes in perfusion. However, since ASL is unavailable as a stock sequence on MRI-linac systems, our objective was to implement the first MRI-linac ASL sequence, characterize its performance, and measure tumour perfusion dynamics.

**Materials and Methods:**

Forty-seven glioblastoma patients were imaged using 3D pseudo-continuous ASL on a 1.5 T MRI-linac during treatment. ASL labeling efficiency was measured in two healthy volunteers and three patients on the MRI-linac and a 1.5 T MR-simulation scanner. ASL cerebral blood flow (CBF) values and repeatability were characterized. Regions of high tumour CBF were evaluated for overlap with the gross tumour volume (GTV) and temporal dynamics.

**Results:**

The labelling efficiency was lower for the MRI-linac compared to the MR-sim and literature consensus values (0.59 vs. 0.88 vs. 0.85). Corrected grey matter CBF was comparable between the MRI-linac and literature (38 ± 13 ml/100 g/min vs. 36.5 ± 8.2 ml/100 g/min, p = 0.41). The within-subject coefficient of variation was similar to literature values (16 % vs. 11 ± 5 %). Across patients, approximately half of the high-CBF region did not overlap the GTV (median 47 %). The high-CBF region tended to decrease in volume during radiotherapy, from − 24 % at week 3 (p = 0.016) to − 45 % (p = 0.044) by week 5 relative to week 1.

**Conclusion:**

MRI-linac ASL could allow targeting and adaptation for highly perfused tumour in future trials for glioblastoma.

## Highlights

• First report of magnetic resonance-guided linear accelerator arterial spin labelling.
• Accurate cerebral blood flow (CBF) required labelling efficiency correction.
• Approximately half the high-CBF tumour was non-enhancing.
• High-CBF tumour decreased in volume by −45 % (median) by week 5 of radiotherapy.

## Graphical abstract

**Figure:**
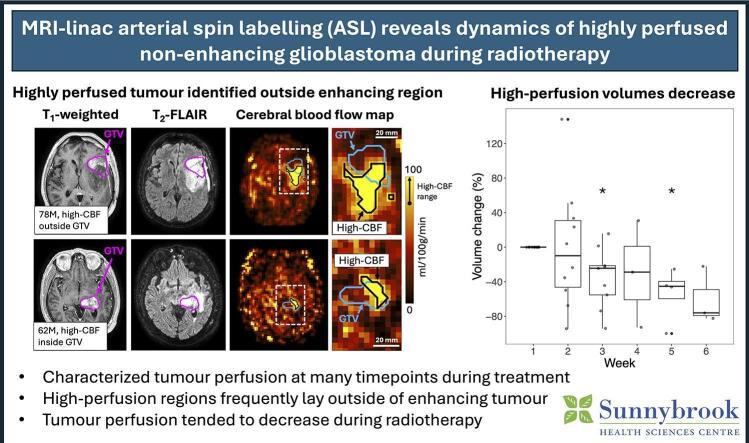

## 1 Introduction

Glioblastomas are among the most vascularized of human tumours [1]. Higher tumour perfusion pre-treatment has been linked to worse patient outcome [[2], [3], [4], [5]], as highly-perfused regions of gliomas may be the regions of greatest grade and aggressiveness [6]. Therefore, highly-perfused tumour regions are currently being investigated as a boost target to potentially extend survival [7]. Targeting such regions adaptively to ensure full coverage despite changes to the vasculature could be even more beneficial. Frequent adaptation is possible using MRI-linear accelerators (MRI-linacs), which combine an MRI scanner and linear accelerator into one device, enabling daily MRI during radiotherapy and adaptation to changes in tumour shape, size, and biology [8]. MRI-linacs are already being used to treat glioblastoma, with adaptation in clinical trials based on gross tumour and cavity changes [9,10]. Perfusion MRI on the MRI-linac could be used to guide treatment and investigate glioblastoma perfusion dynamics with much finer temporal detail than previously feasible.

We aimed to implement a perfusion imaging sequence on our institution’s MRI-linac, both to better understand glioblastoma perfusion dynamics and to develop new strategies for radiotherapy targeting. Conventional imaging of glioblastoma includes contrast-enhanced T_1_-weighted imaging, which shows enhancement in regions of leaky tumour microvasculature, and T_2_-weighted fluid attenuated inversion recovery (FLAIR), which demonstrates regions of hyperintensity surrounding the tumour resulting from edema and tumour infiltration [11,12]. However, contrast enhancement is only visible where vasculature is leaky, while infiltration is difficult to differentiate from edema in T_2_-FLAIR imaging. Perfusion imaging can detect highly perfused non-enhancing or invading tumour which may later develop into gross disease [[13], [14], [15]]. The most common method for perfusion MRI is dynamic susceptibility contrast, which uses gadolinium-based contrast agents; however, this is not ideal, given the concern around repeated injection of gadolinium, the expense of gadolinium-based contrast agents, and risks for patients with kidney failure [16,17]. Hence, we implemented 3D pseudo-continuous arterial spin labelling (PCASL) on our MRI-linac, an alternative method of perfusion imaging that does not rely on exogenous contrast agents.

PCASL uses inversion radiofrequency (RF) pulses to label inflowing water protons in arterial blood at the level of the neck [18]. After a post-label delay (PLD) to allow delivery of the labelled spins to tissue, the brain is imaged to create a “label” image. The inverted spins will partially cancel the effect of the aligned spins in each voxel – the greater the perfusion in a particular voxel, the greater the signal decrease. The imaging sequence is repeated but without the labelling module, to yield a “control” image. The difference between the label and control images is related to the perfusion in each voxel. A common implementation uses a single post-label delay to quantify the cerebral blood flow (CBF), measured in units of millilitres of blood delivered per minute per 100 g of tissue (ml/100 g/min). One parameter that can significantly affect the measured CBF is the labelling efficiency, which quantifies the effective fraction of spins in the arterial blood that are successfully inverted during the labelling module. The modifications made to the scanner in an MRI-linac to accommodate the accelerator may affect the ASL labelling efficiency and therefore the quantification of cerebral blood flow [19,20].

There are no prior reports of arterial spin labelling on the 1.5 T MRI-linac [21,22]. In this paper, we characterize the performance of MRI-linac ASL for measuring blood flow in glioblastomas during radiotherapy. We also describe the temporal changes in perfusion in these tumours throughout a course of radiotherapy.

## 2 Materials and methods

### 2.1 Patients and scanning

Forty-seven glioblastoma patients were treated on a 1.5 T MRI-linac (Unity, Elekta, Sweden) including daily T_1_-weighted imaging and weekly or twice-weekly ASL. All patients participated in a clinical trial using reduced clinical target volume margins and weekly adaptation (NCT04726397, NCT05565521, NCT05720078) [9]. Four healthy volunteers were scanned on the MRI-linac and a 1.5 T MR-simulation scanner (Ingenia, Philips, Netherlands) (“MR-sim”). Patients were immobilized with custom thermoplastic masks during treatment and scanning; volunteers were not immobilized during scans. Written informed consent was obtained from all participants. See Table 1 for patient characteristics.

**Table 1:** Clinical characteristics of patient cohort. The number of patients within each category is listed in the Count/Value column except for Age, for which the median and range are listed.

Characteristic	Category	Count/Value
Age	Median	67
	Range	28–80

Sex	Female	18
	Male	29

MGMT	Methylated	26
	Unmethylated	15
	Unknown / Not assessed	6

Location	Frontal lobe	15
	Occipital lobe	1
	Parietal lobe	8
	Temporal lobe	11
	Other	12

Extent of resection	Biopsy	9
	Gross total	19
	Subtotal	18
	Unknown / Not assessed	1

Dose schedule	40 Gy/15 fx	20
	52.5 Gy/15 fx	3
	54 Gy/30 fx	1
	60 Gy/30 fx	23

ASL is not available by default on the MRI-linac but was enabled using a software patch, through a research agreement with Philips. In all patients, whole-brain pseudo-continuous arterial spin labelling was acquired on the MRI-linac with a 3D gradient and spin echo (GraSE) readout (TR/TE = 4100/16 ms, 4 × 4 × 8 mm^3^ voxels, 8 control-label pairs, label duration = 1800 ms, PLD = 2000 ms, background suppression on, scan time = 10 min. 15 sec.). The acquisition parameters were chosen to match the consensus recommendations for ASL as closely as possible while respecting the limitations of the MRI-linac (e.g., the lower signal-to-noise ratio necessitated large voxels) [23,24]. The labelling plane was placed ∼ 40 mm below the inferior edge of the cerebellum. The imaging volume and labelling plane both had axial alignment. An M_0_ calibration sequence excluding the labelling module and background suppression was also acquired to determine the signal intensity of fully relaxed blood spins, which is necessary for CBF quantification (scan time = 1 min. 26 sec.). Whole-brain ASL was acquired in two volunteers on the MRI-linac (Volunteers 1 and 2) and in one of the same volunteers (Volunteer 1) on the MR-sim. MRI-linac scans used the standard 2 × 4 anterior-posterior coil array [25], while the MR-sim scans used a Philips head coil.

In three patients and two separate volunteers (Volunteers 3 and 4), ASL labelling efficiency, denoted α, was measured on the MRI-linac at the base of the cerebellum using a single-slice Look-Locker echo planar imaging sequence with venous inflow suppression following a labelling module (TR/TE = 46/22 ms, 4 × 4 × 8 mm^3^ voxels, label duration = 1800 ms, PLDs: 20–2042 ms with 46 ms spacing, background suppression off, scan time = 2 min. 48 sec.) [26]. Venous inflow suppression used a spatial saturation band over the head (80 mm thickness) to reduce confounding signal from the jugular veins. The same sequence was also acquired on the MR-sim for the same two volunteers (TR/TE = 32/15 ms, 4 × 4 × 8 mm^3^ voxels, label duration = 1800 ms, PLDs: 20–1430.8 ms with 32.1 ms spacing, scan time = 2 min. 48 sec.). Calibration sequences excluding the labelling module (background suppression was already off) were acquired for quantification (scan time = 1 min.).

### 2.2 Parameter estimation

The signals at each PLD from the left and right internal carotid arteries (ICAs) were collected from the labelling efficiency acquisition using 8 × 8 × 8 mm^3^ regions of interest. The labelling efficiency was estimated from the dynamic ASL signal vs. PLD using a velocity-dependent model, as described in Chen et al. (Supplementary Fig. S1) [26]. The discrete velocity components used in the model included 5 to 40 cm/s in increments of 5 cm/s. The time range used for fitting started at the first signal point when a negative slope began to 450 ms later. For the 3D PCASL sequence, the CBF maps were estimated using a single-PLD model implemented in Oxford_asl [27]. Maps were fitted using both the median measured labelling efficiency across subjects and the labelling efficiency recommended by the consensus paper for ASL (α = 0.85) [23]. The computed CBF is inversely proportional to the labelling efficiency.

### 2.3 Registration and segmentation

The gross tumour volume (GTV) and clinical target volume (CTV) were contoured as part of radiation treatment planning and at weekly adaptive treatment fractions by radiation oncologists, which included recontouring and replanning [9]. The GTV was defined as the surgical cavity plus residual contrast-enhancing tumour while the CTV was a 5 mm expansion plus non-contrast-enhancing tumour as evaluated using T_2_-FLAIR imaging at the discretion of the treating physician, according to the small-margin adaptive trial in which these patients were treated.

Grey matter segmentations were produced from the T_1_-weighted imaging using ANTs [28]. For patients, the tumour was masked out during the segmentation, then the grey matter mask was restricted to the contralateral hemisphere. For healthy volunteers, grey matter was further subdivided into left- and right-hemisphere grey matter for comparison.

The ASL calibration scans were co-registered using the FreeSurfer command mri_robust_template [29]. The same transforms were applied to the CBF maps to align them to the template volume. The T_1_-weighted images were rigidly registered to the aligned ASL scans and resampled to the same geometry. The regions-of-interest were also aligned and resampled using the same transformation.

### 2.4 Grey matter CBF values and repeatability

The median and 95 % quantile of CBF values in grey matter and the CTV were collected from the grey matter segmentations. Median CBF values were compared between scanners for both the measured and literature-recommended labelling efficiencies. CBF values were also compared between the left and right hemispheres for the healthy volunteers with whole-brain ASL. The within-subject standard deviation (wSD) and within-subject coefficient of variation (wCV) of grey matter CBF were estimated from repeated measurements for each subject using a linear mixed-effects model [30]. Patients were included only in this model if they had more than one ASL scan during radiotherapy. Changes in the 95 % quantile of CBF in the CTV were quantified by comparison with the repeatability coefficient of the median grey matter CBF, to determine the number of patients for which a statistically significant change in maximum tumour blood flow could be detected.

### 2.5 High-CBF regions

A region of high-CBF tumour was defined using a thresholding method. First, the median across patients of the 95 % quantile of CBF values in (contralateral) grey matter at baseline was calculated to estimate the maximum grey matter blood flow, $\mathrm{CB}F_{\mathrm{gm},max}$. The “baseline” ASL scan was defined as the earliest ASL scan taken during radiotherapy. All voxels within the treatment planning CTV such that $\mathrm{CBF}>CBF_{\mathrm{gm},max}$ were selected and the largest connected component was taken as the “high-CBF region.”.

At baseline, the proportion of the GTV occupied by the high-CBF region and the proportion of the high-CBF region that did not overlap with the GTV (i.e., lay outside the GTV) were calculated as follows:

$$\text{\%GTV occupied by high-CBF}=\frac{\left|hCBF\cap GTV\right|}{\left|GTV\right|}$$

$$\text{\%High-CBF outside GTV}=\frac{\left|hCBF\cap \neg GTV\right|}{\left|hCBF\right|}$$

where *hCBF* is the high-CBF region, ∩ is the intersection, and $\left|\cdot \right|$ is the volume of the region. Patients were included only if their high-CBF region was greater than 1 cm^3^.

To calculate the dynamics of the high-CBF regions, patients were included only if their baseline ASL scan was during the first week of radiotherapy and they had at least two ASL scans. The high-CBF volume in cm^3^ was calculated for each week after the first week, and the relative percentage change from baseline was calculated as

$$\%\Delta hCBF_{k}=\frac{\mathrm{hCB}F_{k}-hCBF_{1}}{\mathrm{hCB}F_{1}}$$

where $\mathrm{hCB}F_{k}$ is the high-CBF region volume at week k and $\mathrm{hCB}F_{1}$ is the high-CBF region volume at baseline (i.e., week 1).

### 2.6 Statistics

All statistics were done using R version 4.1.2. Comparisons of continuous variables between two groups or to zero were done using t-tests or z-tests. Where a comparison was made to a literature value or a measurement with N = 1, the p-values were calculated using the cumulative distribution function of the Student’s t-distribution or the standard normal distribution. P-values less than 0.05 were considered statistically significant. P-values were not adjusted for multiple comparisons, since this was a hypothesis generating study.

## 3 Results

**Fig. 1:**
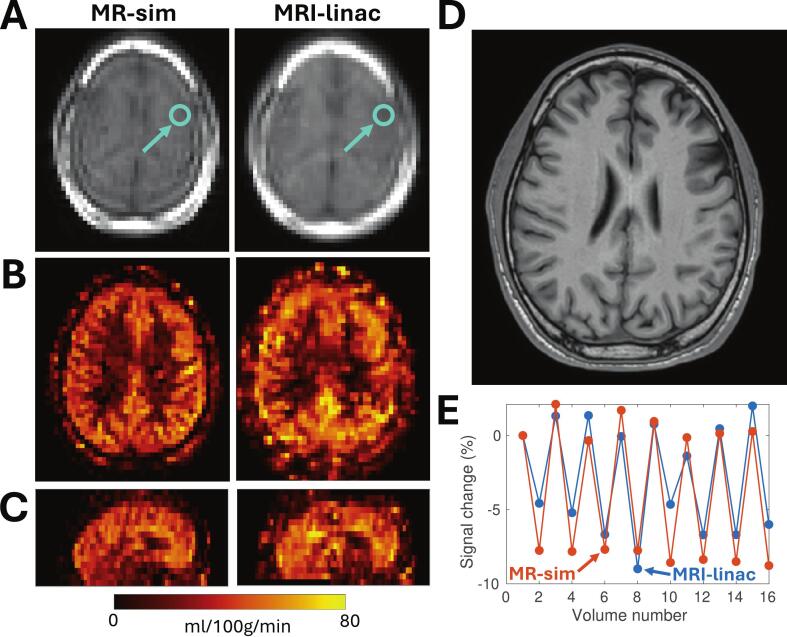
Comparison of MRI-linac and MR-sim ASL in the same healthy volunteer. (A): Axial views of the control acquisition for ASL in one healthy volunteer scanned on both the MRI-linac and MR-sim. (B, C): The corresponding CBF maps in an axial and sagittal view. (D): A T_1_-weighted image in the same slice from the MR-sim is shown for anatomical reference. (E): The average signal versus volume number over an 8 × 8 × 8 mm^3^ region of interest in grey matter (cyan regions in A). The volumes alternate between control and label acquisitions, repeating for eight dynamics. The signal is normalized to the first volume for each scanner. The magnitude of the signal fluctuations is slightly smaller for the MRI-linac (6.7 ± 1.5 percentage points) compared to the MR-sim (8.7 ± 0.9 percentage points), consistent with the lower labelling efficiency (Fig. 2). (For interpretation of the references to color in this figure legend, the reader is referred to the web version of this article.)

**Fig. 2:**
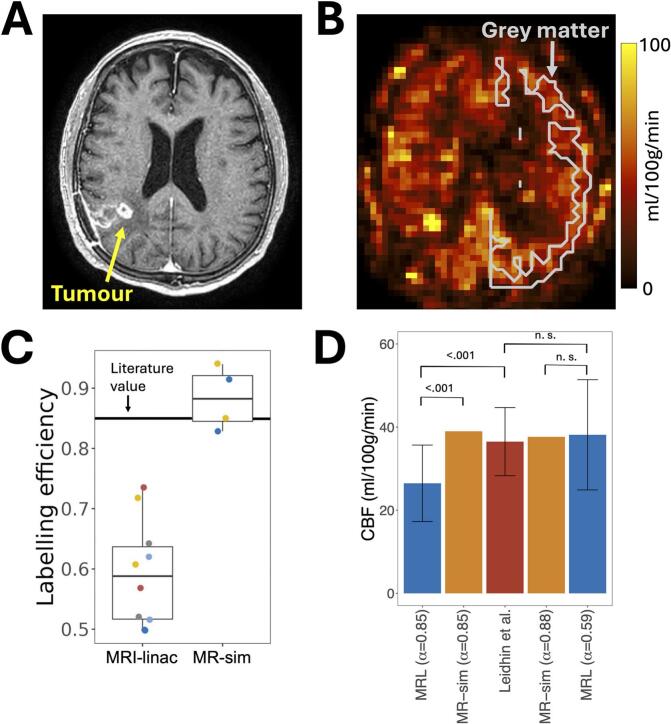
Labelling efficiency correction is required to obtain ASL-derived cerebral blood flow values on an MRI-linac comparable to diagnostic scanners. (A, B): A T_1_-weighted image and a CBF map from one patient showing contralateral grey matter, used for quantifying CBF values and repeatability. (C): The measured MRI-linac labelling efficiency is lower than that of the MR-sim and the literature-recommended value (thick black line) [23]. (D): CBF over grey matter for the MRI-linac (MRL), MR-sim, and from Leidhin et al [32]. The bar heights are means and the error bars are standard deviations across subjects. The values for the MRI-linac and MR-sim were calculated with both the labelling efficiency recommended in literature (α = 0.85) and that measured in this study (α_MRL_ = 0.59, α_MRsim_ = 0.88). Using the literature labelling efficiency results in discrepancy between CBF from the MRI-linac and from standard scanners (p < 0.001), while using the measured labelling efficiency results in no statistically significant differences (n.s., not significant).

See Supplementary Fig. S2 for a flowchart showing the number of participants used per study. The number of participants (N) is indicated with each result below, when non-obvious. Examples of MRI-linac and MR-sim ASL acquisitions and CBF maps are shown in Fig. 1. The MRI-linac and MR-sim labelling efficiencies had median values of 0.59 (range 0.50–0.74, N = 5) and 0.88 (range: 0.83–0.94, N = 2), respectively. Using the literature-recommended labelling efficiency (α = 0.85) to fit CBF maps resulted in a discrepancy between MRI-linac CBF (26 ± 9 ml/100 g/min, N = 47) and CBF as measured on the MR-sim (39 ml/100 g/min, p < 0.001, N = 1) and from literature (36.5 ± 8.2 ml/100 g/min, p < 0.001); using the measured labelling efficiencies, however, there were no detectable differences in grey matter CBF between the MRI-linac (38 ± 13 ml/100 g/min, N = 47) and MR-sim (38 ml/100 g/min, p = 0.80, N = 1) or literature (36.5 ± 8.2 ml/100 g/min, p = 0.41) (Fig. 2). The baseline day for each patient varied, with a median of 6 days from the start of treatment (range: 1–42 days).

CBF was comparable between the left and right hemispheres (Supplementary Fig. S3). There were no statistically significant differences between left and right grey matter CBF values for the first (45 ± 19 vs. 44 ± 19 ml/100 g/min, p = 0.94) and second (37 ± 18 vs. 39 ± 19 ml/100 g/min, p = 0.95) healthy volunteers on the MRI-linac and for the healthy volunteer on the MR-sim (40 ± 12 vs. 36 ± 11 ml/100 g/min, p = 0.82). Similar results were seen for white matter CBF. The wSD of grey matter CBF was 5.6 ml/100 g/min and the wCV was 16 % (N = 33). This repeatability was sufficient to detect changes in maximum tumour CBF in 26/33 (79 %) of participants (Supplementary Fig. S4), with 24/33 (73 %) participants showing decreases in CBF over time.

The 95th percentile of the CBF values in grey matter had a median across patients of 79 ml/100 g/min (N = 47), which served as the threshold to define the high-CBF regions with the clinical target volume. The baseline volume of the high-CBF region across all subjects had a median value of 1.2 cm^3^ (range: 0–93 cm^3^, N = 24) (Supplementary Fig. S5). For the 12 subjects included in the dynamics study, the median baseline high-CBF volume was 5.1 cm^3^ (range: 1.5–41 cm^3^) and the median GTV volume was 22 cm^3^ (range: 4.6–103 cm^3^).

**Fig. 3:**
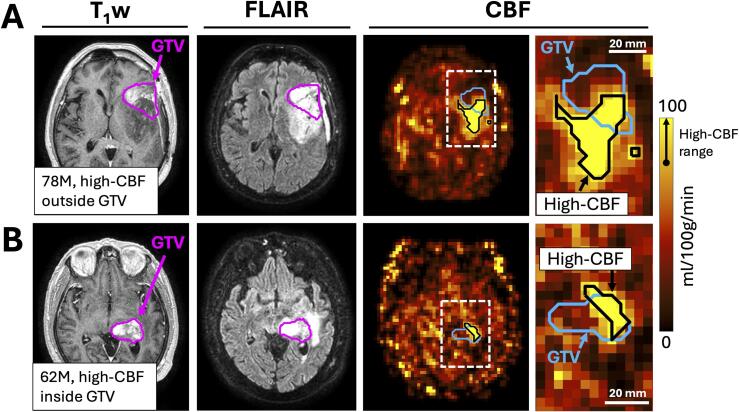
Extension of high-CBF region outside of gross tumour volume. A wide distribution was observed for the proportion of the high-CBF region which lay outside the GTV. (A): An example of a 78-year-old male patient with a large proportion of high-CBF outside the GTV (86 %). The tumour was partially resected and was MGMT unmethylated. From left to right: T_1_-weighted imaging, T_2_-FLAIR imaging, CBF map, zoomed CBF map. The GTV is shown in magenta and in blue for the anatomical images and CBF maps, respectively. The high-CBF region is shown as a black contour on the CBF map. The white dashed box over the CBF map shows the zoomed region. (B): The same set of images for a 62-year-old male patient with a small proportion of high-CBF outside the GTV (20 %). The tumour was biopsied only and was MGMT unmethylated. The color bar shows the range of values that are included in the high-CBF region (>79 ml/100 g/min). (For interpretation of the references to color in this figure legend, the reader is referred to the web version of this article.)

At baseline, a median of 10 % of the GTV exhibited high-CBF (range: 0–50 %, N = 24). Approximately half of the high-CBF region did not overlap with the GTV (median, 47 %; range, 7–100 %, N = 24) (Supplementary Fig. S6). Fig. 3 shows two opposing examples of high-CBF overlap with the GTV to illustrate the variation across patients: one patient with a large proportion of the high-CBF region outside the GTV (86 %) and one patient with a small proportion (20 %).

**Fig. 4:**
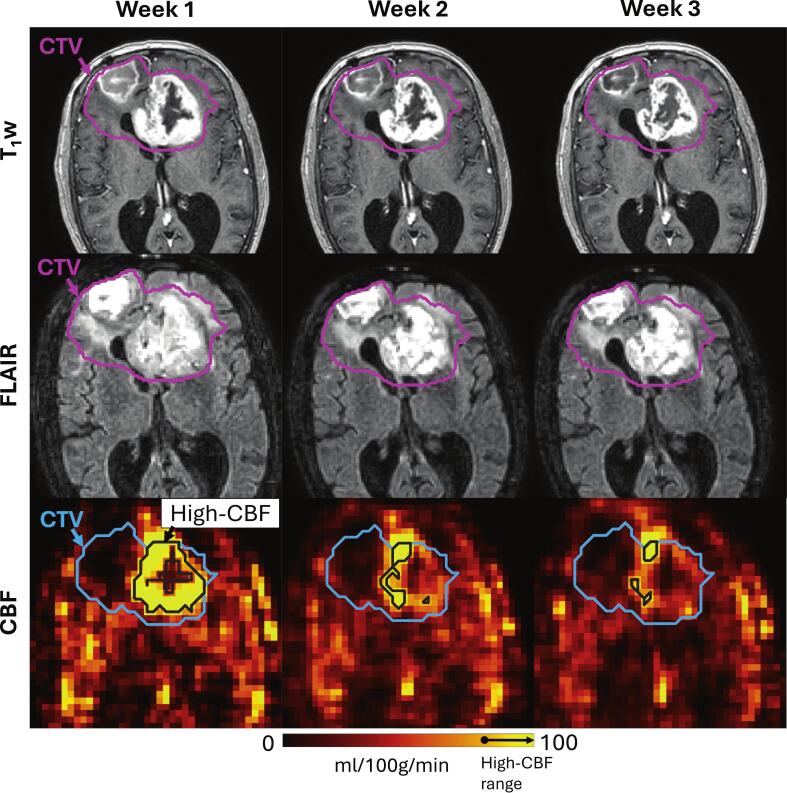
An example of high-CBF region dynamics. T_1_-weighted imaging, T_2_-FLAIR imaging, and CBF maps over three weeks of radiotherapy for a 75-year-old female glioblastoma patient treated with 52.5 Gy/15. The planning CTV is shown in magenta and blue in the anatomical imaging and CBF maps, respectively. The high-CBF region is the black contour on the CBF maps; the arrow on the color bar shows the range of values included in the high-CBF region (>79 ml/100 g/min). The CBF maps reveal substantial decreases in the volume of the high-CBF tumour over time (week 1, 41 cm^3^; week 2, 26 cm^3^; week 3, 11 cm^3^) with minimal change visible in the anatomical imaging. (For interpretation of the references to color in this figure legend, the reader is referred to the web version of this article.)

**Fig. 5:**
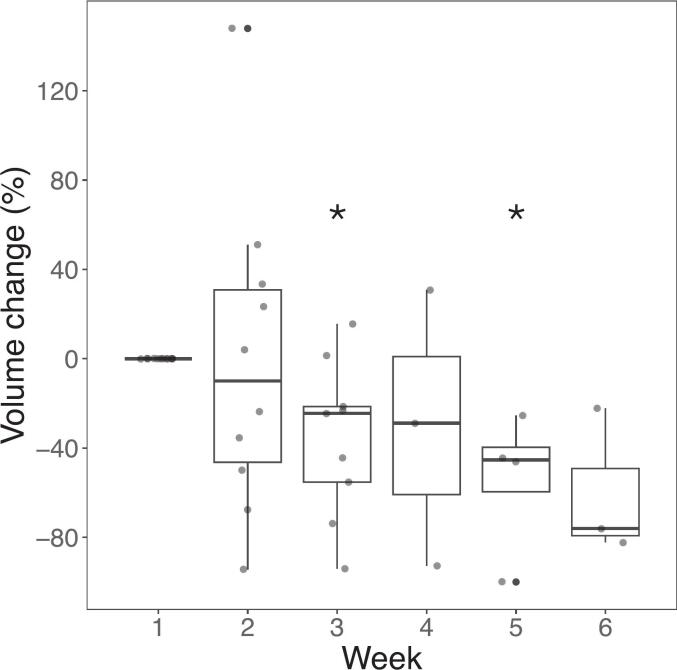
High-CBF volume changes relative to week 1 of radiotherapy. Asterisks indicate statistically significant difference relative to zero change. The high-CBF volume tends to decrease from week to week. Note that some patients included in this graph were treated with 15 fractions and others were treated with 30 fractions.

Fig. 4 shows an example of a patient with substantial decreases in tumour perfusion but minimal change in the appearance of the tumour on anatomical imaging. Decreased perfusion was a trend over the cohort: Across the 12 subjects included in the dynamics study, the high-CBF volume decreased during radiotherapy from a median change of 0 % at week 1 (by definition) to − 24 % at week 3 (p = 0.016) to − 45 % (p = 0.044) by week 5 (Fig. 5). High-CBF time series for individual participants are shown in Supplementary Fig. S7.

## 4 Discussion

We implemented an arterial spin labelling protocol on the 1.5 T MRI-linac and used it to quantify perfusion dynamics in glioblastoma during radiotherapy. The novelty of the current study is the frequent ASL acquisition during radiotherapy – at least once per week and often twice per week – which allows quantification of the dynamics of highly perfused regions over time that has not been previously described. We found that MRI-linac CBF values were comparable to those from diagnostic scanners after labelling efficiency correction, and that tumour regions of high CBF extended outside the enhancing tumour and decreased in size during radiotherapy.

The labelling efficiency of MRI-linac ASL was lower than expected based on previous literature and the cause is unclear. The labelling module for the ASL pulse sequence for the MRI-linac and the MR-sim used in this study are identical. The gradient non-linearities or eddy current fields at the level of each subject’s neck on each scanner could be different, which would lead to a discrepancy between labelling efficiency values [26,31]. Regardless of the cause, once CBF values were corrected for the true labelling efficiency, the CBF values obtained in contralateral grey matter from the MRI-linac were similar to those from diagnostic-quality scanners [32]. Additionally, the repeatability as measured by the wCV (16 %) was comparable to that previously reported (11 ± 5 %) [33]. While the baseline scan timepoint at which grey matter CBF was calculated showed a range of values, this was expected to have a minimal impact, since there is little change in CBF in normal tissue during radiotherapy [34].

Highly perfused tumour regions were defined as parts of the CTV with a CBF value above the “maximum” CBF (the 95 % quantile) of contralateral grey matter. The threshold was chosen to isolate active perfused tumour from cavity, normal tissue, etc. Admittedly, the threshold is arbitrary, and a sensitivity analysis correlating high-CBF changes with patient outcome for varying thresholds should be done once patient outcomes are mature.

Highly perfused tumour regions were consistently found outside of the contrast-enhancing tumour, consistent with the findings of other authors [4]. Perfusion imaging may reveal components of the tumour not captured by conventional anatomical imaging in certain cases. The implications for treatment planning could be substantial, as this finding implies that not all the active tumour is adequately covered by the GTV as currently defined. The high-perfusion dynamics showed decreased size of the high-CBF region over time during radiotherapy. Additionally, the maximum tumour CBF also tended to decrease. These findings were consistent with previous literature that shows decreased perfusion following radiotherapy relative to pre-radiotherapy and the known effect of radiation injury to blood vessels [35,36]. A handful of other studies have acquired perfusion imaging at select timepoints during radiotherapy, reporting complex dynamics [2,34].

This work had several limitations: the number of patients was small for both the labelling efficiency and dynamics studies; the large voxel size of the ASL protocol (4 × 4 × 8 mm^3^) may have affected the measurements of cerebral blood flow through partial volume averaging; and the geometric distortions present in the GraSE images because of the echo-planar readouts could have caused misalignment with the T_1_-weighted images used to define regions of interest. For the dynamics analysis, we only evaluated the effect of time and did not account for differing fractionation schedules and accumulated dose; this was necessary because of the small number of patients. In future work, we will examine the effects of misregistration or partial volume averaging on the CBF results. Determining a sequence with lower distortion is desirable.

Future work will include incorporating ASL alongside diffusion-weighted imaging (DWI) for early response prediction [37,38]. Low diffusion often indicates a cell-dense, non-necrotic region of tumour [39,40], while larger highly-perfused regions predict worse survival [2,3]. Therefore, a region of high perfusion and low diffusion could be used to predict future regions of tumour recurrence [4,41], and therefore identify which regions of hyperintensity on T_2_-FLAIR should be included in the CTV.

## CRediT authorship contribution statement

**Liam S.P. Lawrence:** Conceptualization, Methodology, Software, Formal analysis, Writing – original draft, Writing – review & editing. **Brige P. Chugh:** Conceptualization, Methodology, Writing – review & editing. **James Stewart:** Data curation, Writing – review & editing. **Mark Ruschin:** Supervision, Writing – review & editing. **Aimee Theriault:** Data curation, Writing – review & editing. **Jay Detsky:** Supervision, Resources, Writing – review & editing. **Pejman J. Maralani:** Methodology, Supervision, Resources, Writing – review & editing. **Chia-Lin Tseng:** Supervision, Resources, Writing – review & editing. **Hany Soliman:** Supervision, Resources, Writing – review & editing. **Mary Jane Lim-Fat:** Supervision, Resources, Writing – review & editing. **Sunit Das:** Supervision, Resources, Writing – review & editing. **Arjun Sahgal:** Supervision, Resources, Project administration, Funding acquisition, Writing – review & editing. **Angus Z. Lau:** Conceptualization, Methodology, Supervision, Resources, Project administration, Funding acquisition, Writing – review & editing.

## Declaration of competing interest

The authors declare the following financial interests/personal relationships which may be considered as potential competing interests: L.S.P.L.: Received travel expenses/accommodations from Elekta AB. B.P.C.: Previously held an industry partnership grant with Modus QA. J.S.: None. M.R.: Co-inventor of and owns associated intellectual property specific to the image-guidance system on the Gamma Knife Icon. None related to this work. A.T.: None. J.D.: None. P.J.M.: None. C.-L.T..: Travel accommodations/expenses & honoraria for past educational seminars by Elekta; belongs to the Elekta MR-Linac Research Consortium; advisor/consultant with Sanofi. H.S.: None. M.J.L.-F.: None. S.D.: Receives funding from CIHR, the Canadian Cancer Society, and the Keenan Chair in Surgery. He serves as the Provincial Lead for CNS Cancers at Cancer Care Ontario. His laboratory receives research support from Alkermes. He is on the advisory board of the Subcortical Surgery Group and XPan Medical. A.S.: Elekta/Elekta AB: research grant, consultant, honorarium for past educational seminars, travel expenses; Varian: honorarium for past educational seminars; BrainLab: research grant, consultant, honorarium for educational seminars, travel expenses; AstraZeneca: honorarium for educational seminars; ISRS: President of the International Stereotactic Radiosurgery Society (ISRS); Seagen Inc: honorarium for education seminars and research grants; Cerapedics: honorarium for educational seminars, travel expenses; CarboFIX: honorarium for educational seminars; Servier: honorarium for educational seminars. A.Z.L.: None.

## References

[1] Hardee M.E., Zagzag D. (2012). Mechanisms of glioma-associated neovascularization. Am J Pathol.

[2] Cao Y., Tsien C.I., Nagesh V., Junck L., Ten Haken R., Ross B.D. (2006). Clinical investigation survival prediction in high-grade gliomas by MRI perfusion before and during early stage of RT. Int J Radiat Oncol.

[3] Law M., Young R.J., Babb J.S., Peccerelli N., Chheang S., Gruber M.L. (2008). Gliomas: predicting time to progression or survival with cerebral blood volume measurements at dynamic susceptibility-weighted contrast-enhanced perfusion MR imaging. Radiology.

[4] Wahl D.R., Kim M.M., Aryal M.P., Hartman H., Lawrence T.S., Schipper M.J. (2018). Combining perfusion and high B-value diffusion MRI to inform prognosis and predict failure patterns in glioblastoma. Int J Radiat Oncol.

[5] Leon S.P., Folkerth R.D. (1996). Black PMcL. Microvessel density is a prognostic indicator for patients with astroglial brain tumors. Cancer.

[6] Hirai T., Murakami R., Nakamura H., Kitajima M., Fukuoka H., Sasao A. (2008). Prognostic value of perfusion MR imaging of high-grade astrocytomas: long-term follow-up study. AJNR Am J Neuroradiol.

[7] Kim M.M., Sun Y., Aryal M.P., Parmar H.A., Piert M., Rosen B. (2021). A phase 2 study of dose-intensified chemoradiation using biologically based target volume definition in patients with newly diagnosed glioblastoma. Int J Radiat Oncol.

[8] Otazo R., Lambin P., Pignol J.-P., Ladd M.E., Schlemmer H.-P., Baumann M. (2021). MRI-guided radiation therapy: an emerging paradigm in adaptive radiation oncology. Radiology.

[9] Detsky J., Chan A.W., Palhares D.M., Hudson J.M., Stewart J., Chen H. (2024). MR-linac on-line weekly adaptive radiotherapy for High Grade Glioma (HGG): results from the UNITED single arm phase II trial. Int J Radiat Oncol Biol Phys.

[10] Cullison K., Samimi K., Bell J.B., Maziero D., Valderrama A., Breto A.L. (2025). Dynamics of daily glioblastoma evolution during chemoradiation therapy on the 0.35T magnetic resonance imaging-linear accelerator. Int J Radiat Oncol.

[11] Wen P.Y., Macdonald D.R., Reardon D.A., Cloughesy T.F., Sorensen A.G., Galanis E. (2010). Updated response assessment criteria for high-grade gliomas: response assessment in neuro-oncology working group. J Clin Oncol.

[12] Watanabe M., Tanaka R., Takeda N. (1992). Magnetic resonance imaging and histopathology of cerebral gliomas. Neuroradiology.

[13] Price S.J., Young A.M.H., Scotton W.J., Ching J., Mohsen L.A., Boonzaier N.R. (2016). Multimodal MRI can identify perfusion and metabolic changes in the invasive margin of glioblastomas. J Magn Reson Imaging JMRI.

[14] Price S.J., Green H.A.L., Dean A.F., Joseph J., Hutchinson P.J., Gillard J.H. (2011). Correlation of MR relative cerebral blood volume measurements with cellular density and proliferation in high-grade gliomas: an image-guided biopsy study. Am J Neuroradiol.

[15] Blasel S., Franz K., Ackermann H., Weidauer S., Zanella F., Hattingen E. (2011). Stripe-like increase of rCBV beyond the visible border of glioblastomas: site of tumor infiltration growing after neurosurgery. J Neurooncol.

[16] Hellman R.N. (2011). Gadolinium-induced nephrogenic systemic fibrosis. Semin Nephrol.

[17] Aime S., Caravan P. (2009). Biodistribution of gadolinium-based contrast agents, including gadolinium deposition. J Magn Reson Imaging.

[18] Chappell M., MacIntosh B., Okell T. (2017).

[19] Raaymakers B.W., Lagendijk J.J.W., Overweg J., Kok J.G.M., Raaijmakers A.J.E., Kerkhof E.M. (2009). Integrating a 1.5 T MRI scanner with a 6 MV accelerator: proof of concept. Phys Med Biol.

[20] Tijssen R.H.N., Philippens M.E.P., Paulson E.S., Glitzner M., Chugh B., Wetscherek A. (2019). MRI commissioning of 1.5T MR-linac systems – a multi-institutional study. Radiother Oncol.

[21] Lawrence L.S.P., Chugh B., Stewart J., Ruschin M., Theriault A., Detsky J. (2024). 2024 ISMRM Annu. Meet.

[22] Lawrence L.S.P., Chugh B., Stewart J., Ruschin M., Theriault A., Detsky J. (2025). Improving accuracy of MR-Linac arterial spin labelling for imaging dynamics of highly-perfused tumour regions in glioblastoma. 2025 ISMRM Annu. Meet., Honolulu, Hawaii.

[23] Alsop D.C., Detre J.A., Golay X., Günther M., Hendrikse J., Hernandez-Garcia L. (2015). Recommended implementation of arterial spin-labeled perfusion MRI for clinical applications: a consensus of the ISMRM perfusion study group and the European consortium for ASL in dementia: recommended implementation of ASL for clinical applications. Magn Reson Med.

[24] Lindner T., Bolar D.S., Achten E., Barkhof F., Bastos-Leite A.J., Detre J.A. (2023). Current state and guidance on arterial spin labeling perfusion MRI in clinical neuroimaging. Magn Reson Med.

[25] Hoogcarspel S.J., Zijlema S.E., Tijssen R.H.N., Kerkmeijer L.G.W., Jürgenliemk-Schulz I.M., Lagendijk J.J.W. (2018). Characterization of the first RF coil dedicated to 1.5 T MR guided radiotherapy. Phys Med Biol.

[26] Chen Z., Zhang X., Yuan C., Zhao X., Van Osch M.J.P. (2017). Measuring the labeling efficiency of pseudocontinuous arterial spin labeling. Magn Reson Med.

[27] Chappell M.A., Groves A.R., Whitcher B., Woolrich M.W. (2009). Variational Bayesian inference for a nonlinear forward model. IEEE Trans Signal Process.

[28] Avants B.B., Tustison N.J., Wu J., Cook P.A., Gee J.C. (2011). An open source multivariate framework for n-tissue segmentation with evaluation on public data. Neuroinformatics.

[29] Reuter M., Schmansky N.J., Rosas H.D., Fischl B. (2012). Within-subject template estimation for unbiased longitudinal image analysis. Neuroimage.

[30] Raunig D.L., McShane L.M., Pennello G., Gatsonis C., Carson P.L., Voyvodic J.T. (2015). Quantitative imaging biomarkers: a review of statistical methods for technical performance assessment. Stat Methods Med Res.

[31] Dai W., Garcia D., De Bazelaire C., Alsop D.C. (2008). Continuous flow‐driven inversion for arterial spin labeling using pulsed radio frequency and gradient fields. Magn Reson Med.

[32] Leidhin C.N., McMorrow J., Carey D., Newman L., Williamson W., Fagan A.J. (2021). Age-related normative changes in cerebral perfusion: data from the Irish Longitudinal Study on Ageing (TILDA). Neuroimage.

[33] Mutsaerts H.J.M.M., Steketee R.M.E., Heijtel D.F.R., Kuijer J.P.A., Van Osch M.J.P., Majoie C.B.L.M. (2014). Inter-vendor reproducibility of pseudo-continuous arterial spin labeling at 3 tesla. PLoS One.

[34] Zhou L., Udayakumar D., Wang Y., Pinho M.C., Wagner B.C., Youssef M. (2024). Repeatability and reproducibility of pseudo-continuous arterial spin labeling measured brain perfusion in healthy volunteers and glioblastoma patients. Am J Neuroradiol.

[35] O’Connor M.M., Mayberg M.R. (2000). Effects of radiation on cerebral vasculature: a review. Neurosurgery.

[36] Larsson C., Groote I., Vardal J., Kleppestø M., Odland A., Brandal P. (2020). Prediction of survival and progression in glioblastoma patients using temporal perfusion changes during radiochemotherapy. Magn Reson Imaging.

[37] Lawrence L.S.P., Chan R.W., Chen H., Stewart J., Ruschin M., Theriault A. (2023). Diffusion-weighted imaging on an MRI-linear accelerator to identify adversely prognostic tumour regions in glioblastoma during chemoradiation. Radiother Oncol.

[38] Lawrence L.S.P., Chugh B., Stewart J., Ruschin M., Theriault A., Detsky J. (2024). 2024 ISMRM Annu. Meet.

[39] Ross B.D., Chenevert T.L., Kim B., Ben-Yoseph O. (1994). Magnetic resonance imaging and spectroscopy: application to experimental neuro-oncology. Q Magn Reson Biol Med.

[40] Chenevert T.L., Stegman L.D., Taylor J.M.G., Robertson P.L., Greenberg H.S., Rehemtulla A. (2000). Diffusion magnetic resonance imaging: an early surrogate marker of therapeutic efficacy in brain tumors. J Natl Cancer Inst.

[41] Moore-Palhares D., Lawrence L.S.P., Myrehaug S., Stewart J., Detsky J., Tseng C.-L. (2025). Temporal apparent diffusion coefficient (ADC) changes during chemoradiation: an imaging biomarker for tumour response monitoring and spatial recurrence prediction in glioblastoma. Int J Radiat Oncol Biol Phys.

