# Supplementary material for: Magnetic resonance imaging-guided linear accelerator arterial spin labelling reveals dynamics of highly perfused non-enhancing glioblastoma during radiotherapy
Source: Phys Imaging Radiat Oncol. 2025 Nov 21;36:100870. doi: 10.1016/j.phro.2025.100870 (PMC12702226; doi:10.1016/j.phro.2025.100870)
Supplement: Supplementary Data 1 [file mmc1.pdf]

Supplementary Materials

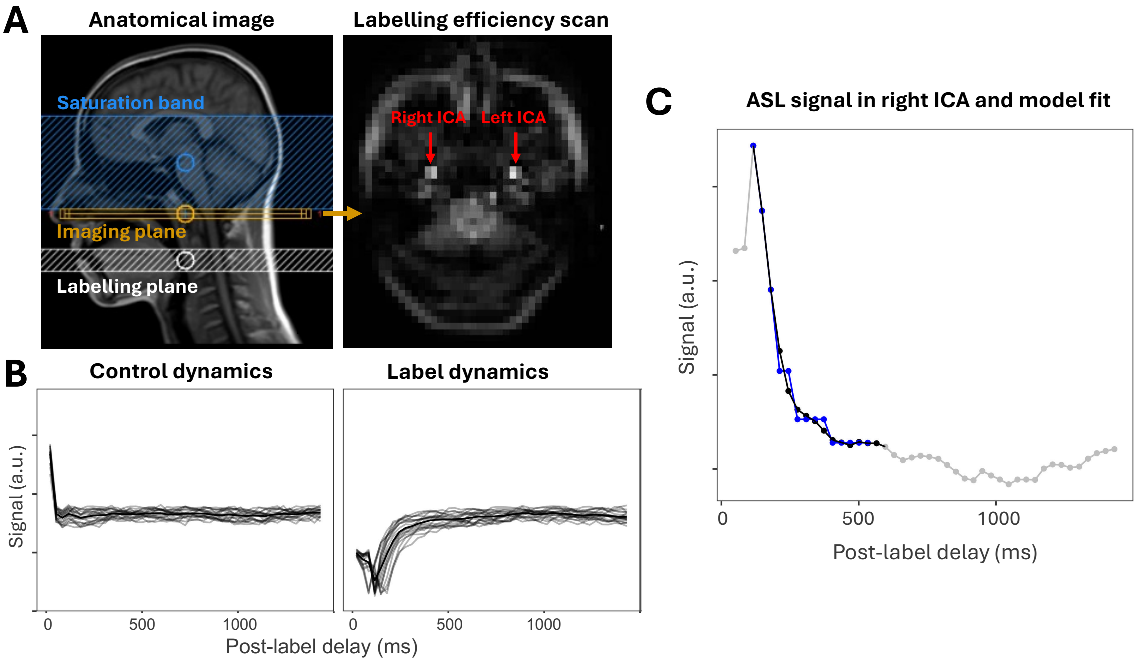

Supplementary Figure S1 – Example of labelling efficiency acquisition. *A single-slice Look-Locker sequence was acquired at the inferior edge of the cerebellum after a labelling module. (**A**): An example of the image planning and a single dynamic of the scan. The right and left internal carotid arteries (ICAs) are indicated. The signal dynamics in the ICAs with and without labelling (label vs. control) were used to estimate an average ASL signal versus post-label delay. (**B**): An example of the signal dynamics for the right ICA. (**C**): Plot of the signal versus post-label delay, showing the ASL signal (control minus label) and the fit to a velocity-dependent boxcar model to determine the labelling efficiency, as described in Chen et al., MRM, 2017 (https://doi.org/10.1002/mrm.26266).*

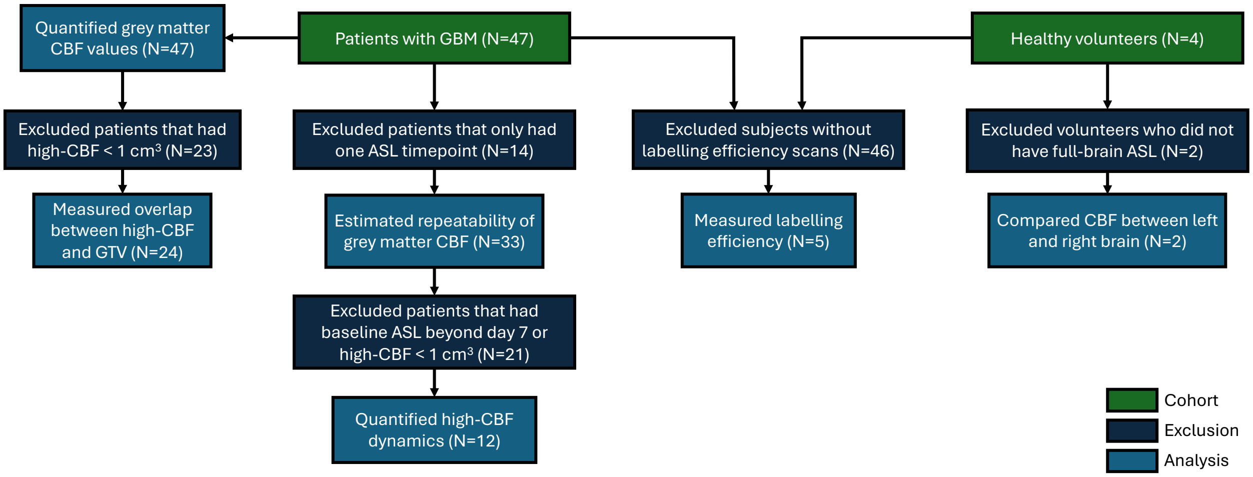

Supplementary Figure S2 – Flowchart showing subjects used in each analysis. *The number of subjects for the patient and volunteer cohorts are shown in the green boxes. Following the arrows shows reasons for excluding certain patients (dark purple boxes) and the number of patients N included in each analysis (blue boxes). Abbreviations: GBM, glioblastoma; ASL, arterial spin labelling; CBF, cerebral blood flow; GTV, gross tumour volume.*

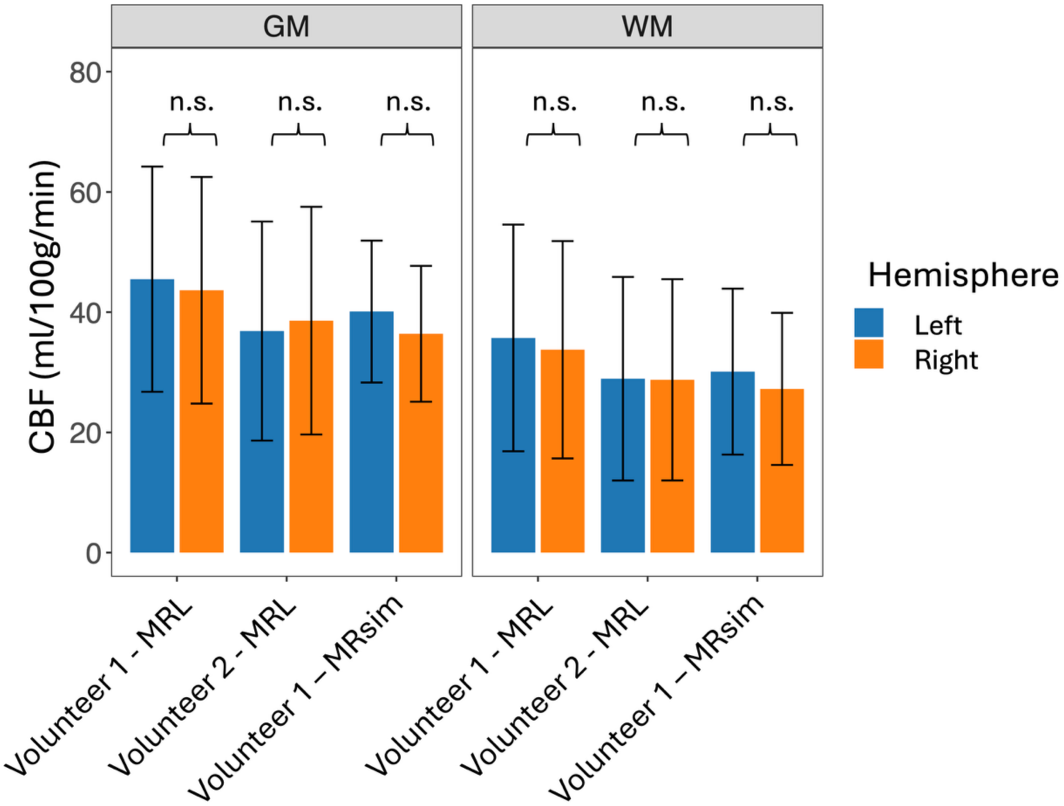

Supplementary Figure S3 – Comparison of cerebral blood flow between left and right hemispheres. *The mean (columns) and standard deviation (error bars) for the CBF in left-brain and right-brain grey matter (GM) and white matter (WM) for healthy volunteers (two on the MR-Linac, one on the MR-sim). The values are comparable between left and right brain in each case (n.s.: not significantly different).*

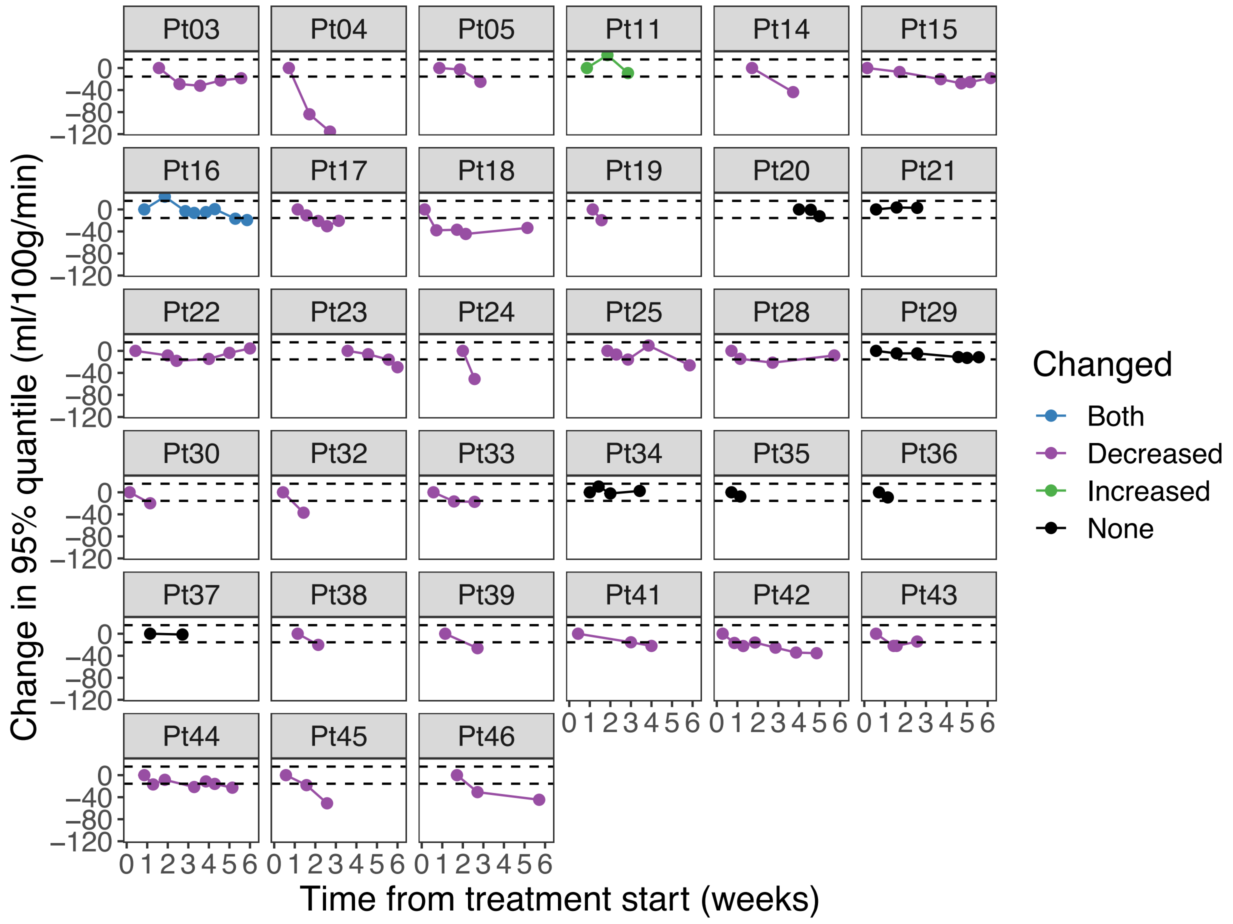

Supplementary Figure S4 – Change in maximum tumour CBF by participant. *The 95% quantile of the CBF over the CTV is plotted as a function of time from treatment start in weeks. The points are measurements and the lines are for visual aid. Each panel is a different participant, labelled by patient number. Only participants with more than one ASL scan during treatment were included (N=33). The dashed black lines indicate the threshold for statistically significant change (the repeatability coefficient of median grey matter CBF). The colour of the plots indicates whether maximum tumour CBF increased, decreased, did both, or did neither. Change was detected for 26/33 (79%) of participants.*

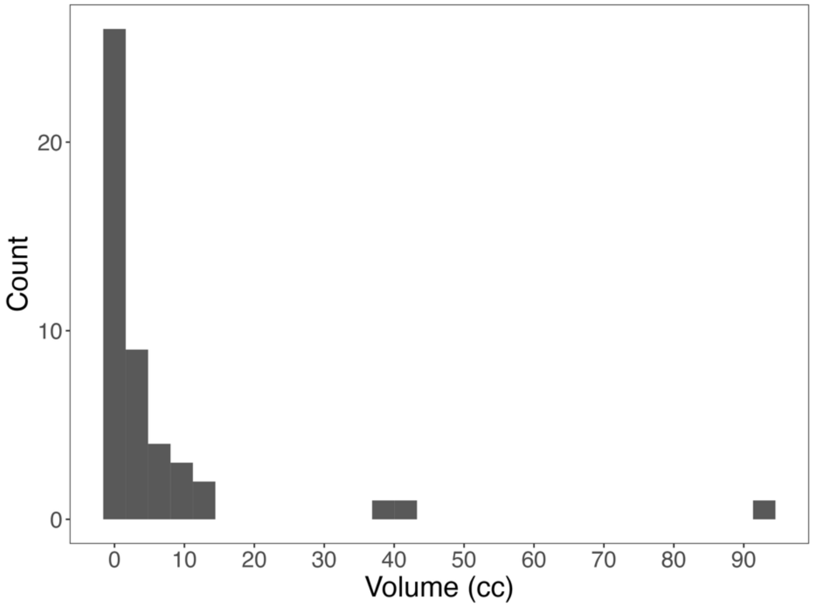

Supplementary Figure S5 – Baseline volume of the high-CBF region across all subjects.

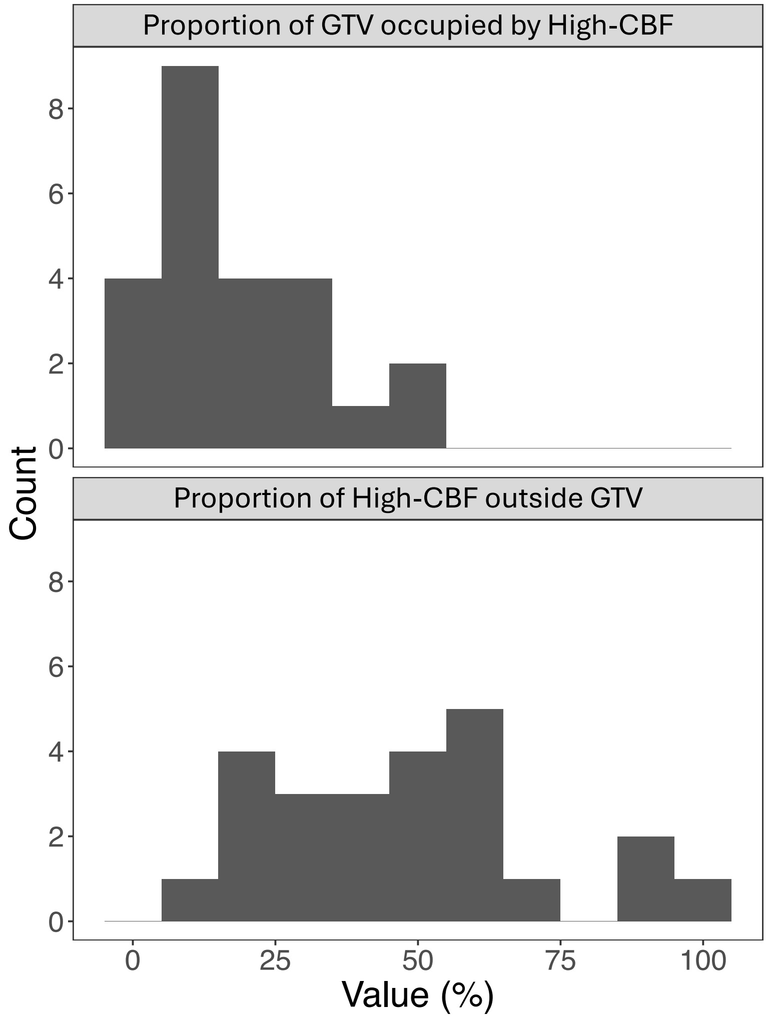

Supplementary Figure S6 – Proportion of the GTV occupied by the high-CBF region and the proportion of the high-CBF region outside of the GTV.

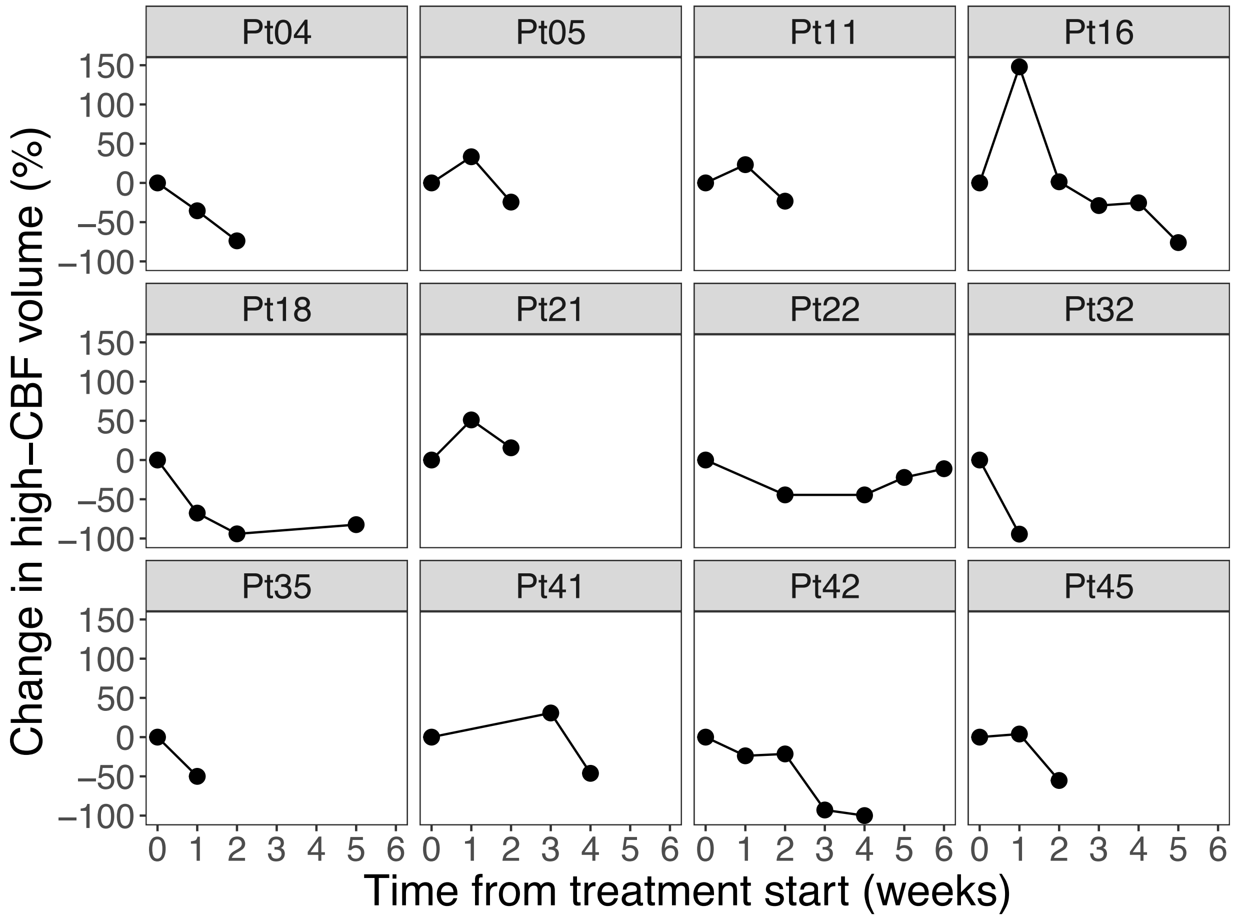

Supplementary Figure S7 – Change in the high-CBF volume per participant. *The change in the high-CBF region relative to the first week of treatment is plotted for those participants who had both an ASL scan within the first week of treatment and multiple ASL scans (N=12). The points are measurements and the lines are for visual aid.*
